# Molecular Imaging and Non-molecular Imaging of Atherosclerotic Plaque Thrombosis

**DOI:** 10.3389/fcvm.2021.692915

**Published:** 2021-07-05

**Authors:** Bingchen Guo, Zhaoyue Li, Peiyang Tu, Hao Tang, Yingfeng Tu

**Affiliations:** ^1^Department of Cardiology, The Second Affiliated Hospital of Harbin Medical University, Harbin, China; ^2^Department of Cardiology, The First Affiliated Hospital of Harbin Medical University, Harbin, China; ^3^College of Clinical Medicine, Hubei University of Science and Technology, Xianning, China

**Keywords:** atherosclerosis, thrombosis, molecular imaging, non-molecular imaging, atherosclerotic plaque thrombosis

## Abstract

Thrombosis in the context of atherosclerosis typically results in life-threatening consequences, including acute coronary events and ischemic stroke. As such, early detection and treatment of thrombosis in atherosclerosis patients is essential. Clinical diagnosis of thrombosis in these patients is typically based upon a combination of imaging approaches. However, conventional imaging modalities primarily focus on assessing the anatomical structure and physiological function, severely constraining their ability to detect early thrombus formation or the processes underlying such pathology. Recently, however, novel molecular and non-molecular imaging strategies have been developed to assess thrombus composition and activity at the molecular and cellular levels more accurately. These approaches have been successfully used to markedly reduce rates of atherothrombotic events in patients suffering from acute coronary syndrome (ACS) by facilitating simultaneous diagnosis and personalized treatment of thrombosis. Moreover, these modalities allow monitoring of plaque condition for preventing plaque rupture and associated adverse cardiovascular events in such patients. Sustained developments in molecular and non-molecular imaging technologies have enabled the increasingly specific and sensitive diagnosis of atherothrombosis in animal studies and clinical settings, making these technologies invaluable to patients' health in the future. In the present review, we discuss current progress regarding the non-molecular and molecular imaging of thrombosis in different animal studies and atherosclerotic patients.

## Introduction

**Figure 1:**
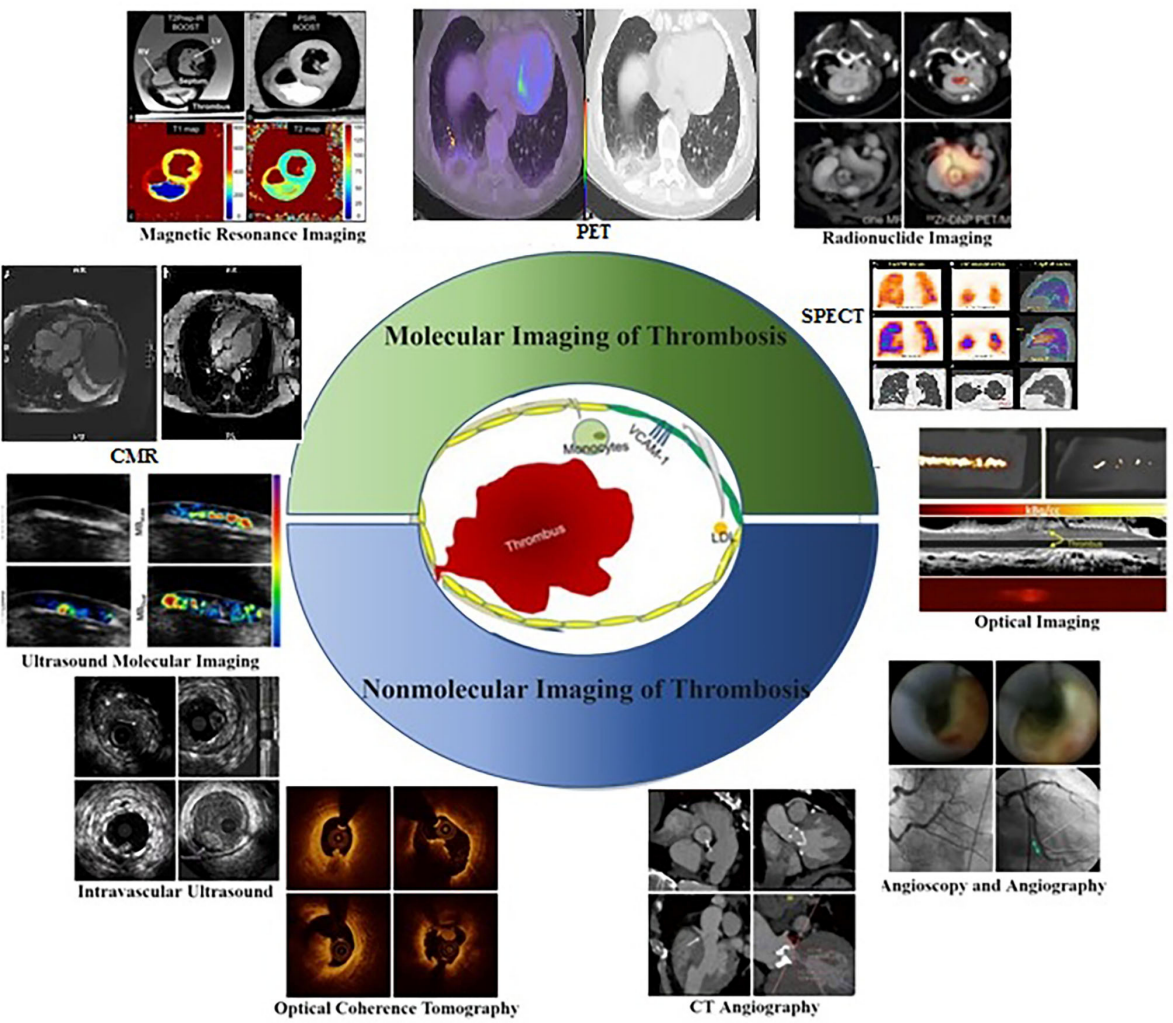
Molecular and Non-molecular thrombosis imaging strategies in atherosclerosis patients for assessing pathophysiological features related to early thrombosis at the molecular and cellular level (13), including ultrasound molecular imaging (6), magnetic resonance imaging (7), radionuclide imaging (14), optical imaging (15), intravascular ultrasound, optical coherence tomography, CT angiography (16), angioscopy and angiography (17).

Platelet surface receptor interactions initiate thrombosis in atherosclerosis. The GPIIb/IIIa collagen receptors on platelets can adhere to fibrillar collagen firmly, and adenosine diphosphate (ADP) further promotes fibrinogen binding and platelet aggregation (1). In addition, the coagulation factor XIIIa plays an essential role in strengthening fibrin cross-linking and thrombin cleavage in thrombosis (2). Thus, these representative receptors serve as molecular imaging targets in the context of thrombosis in atherosclerosis (3). Molecular imaging strategies were first pioneered in 1999 and employ molecular biology and medical imaging strategies to quantitatively analyze dynamic biological processes *in vivo* (4). Relative to traditional imaging to assess disease based on morphological and physiological function changes, these modalities focus on assessing anatomical morphology and physiology and provide significantly more insight into disease's molecular and cellular basis. Molecular imaging strategies rely on the use of highly specific and sensitive targeted probes together with high-resolution imaging equipment to visualize pathological processes such as thrombosis in real-time, enabling clinicians and researchers to better understand the pathophysiological features of early thrombosis (5). Major imaging modalities developed to date include ultrasound (6), magnetic resonance imaging (MRI) (7), positron emission tomography (PET) (8), single-photon emission computed tomography (SPECT) (9), computed tomography (CT) (10), optical imaging (11), and multimodal imaging (12) (Figure 1). The molecular imaging of thrombosis is a highly sensitive and specific approach to guide the diagnosis and treatment of cardiovascular disease (18, 19). Additionally, non-molecular imaging can provide important insights regarding thrombus formation, and plaque vulnerability (20), thereby aiding in the prevention of thrombosis in atherosclerotic patients. Non-molecular imaging strategies rely on the quantification of physiological and pathological processes and the morphological and structural levels. This review is focused on highlighting different non-molecular and molecular imaging techniques and their application for the detection and assessment of Atherosclerotic plaque thrombosis in animal models and clinical settings.

## Non-Molecular and Molecular Imaging Modalities

Since Dr. F. Mason Sones Jr. performed the first selective coronary angiogram by accident in 1958, invasive contrast X-ray angiography has been the gold standard for imaging of coronary atherosclerosis over the past 50 years (21). While remaining the imaging standard and the most studied in regards to patient outcomes, coronary angiography does not provide imaging of atherosclerosis itself, but rather its end result. Major non-molecular imaging techniques include intravascular ultrasound (IVUS), optical coherence tomography (OCT), combined IVUS/OCT imaging, computed tomography angiography (CTA), cardiovascular magnetic resonance (CMR), angioscopy, and angiography (Figure 1). The advent of intravascular ultrasound (IVU) and subsequent intravascular imaging modalities, such as OCT and near-infrared spectroscopy (NIRS) delivered the ability to directly image the vessel wall and atherosclerotic plaque. These intravascular imaging modalities have progressed our understanding of atherosclerosis significantly and have helped to image plaque at all stages of its development, while also defining its compositional features that are associated with plaque vulnerability (22). Although invasive, they have also enabled a means by which to serially monitor the natural history of plaque and its modulation with anti-atherosclerotic therapies. While having the ability to identify vulnerable characteristics, such as high plaque volume, thin fibrous cap, lipid-rich core, spotty calcification, and intraplaque neovascularization and hemorrhage, they are as yet unable to determine the activity of plaque, in terms of pathogenic molecular pathways (23). Ideal modalities for imaging of coronary atherosclerosis should combine non-invasiveness so that patients can be assessed at repeat intervals with minimum risk, and the accurate and reproducible ability to identify early signals of plaque vulnerability that predict a high risk of progression and complication. In addition to conventional structural imaging modalities, such as ultrasonography and computed tomography, molecular imaging with different tracers has been studied for the diagnosis of venous thrombosis (24).

Molecular imaging was christened as a term in the mid to late 1990s to encompass *in vivo* functional imaging modalities, which go beyond anatomical tissue assessment to also visualize and quantify specific biological processes down to a cellular and molecular level. The early progress and focus of development centered on imaging in oncology but has expanded to use throughout medicine (25). Molecular imaging strategies rely on the use of highly specific and sensitive targeted probes together with high-resolution imaging equipment to visualize pathological processes such as thrombosis in real-time, enabling clinicians and researchers to better understand the pathophysiological features of early thrombosis. The various imaging modalities available for the investigation of suspected CAD can be broadly divided into the categories given in below Table 1.

**Table 1:** Various imaging modalities available for the investigation of suspected CAD.

**Invasive techniques (conventional/non-molecular)**	**Non-invasive imaging techniques (advanced/molecular)**
a. Invasive coronary angiography (the traditional gold standard) b. Fractional flow reserve (FFR) c. Intravascular ultrasound and optical coherence tomography.	d. Direct visualization of the coronary arteries: • Coronary calcium score (CAC) • Coronary CT using electron beam CT (EBCT) or multidetector CT (MDCT) • Magnetic resonance angiography of the coronary arteries. e. Assessment of the functional significance of coronary stenosis: • Myocardial perfusion scintigraphy, which includes single-photon emission CT (SPECT) and positron emission tomography (PET) • Stress echocardiography (SE) • Cardiac MRI (CMR) including stress CMR and delayed enhancement sequences.

There are a large number of different molecular imaging agents that can target a diverse range of biological activity across the spectrum of pathologies in medicine. These typically combine a targeting component, that ideally interacts specifically with the biochemical process being investigated, and an imaging component that can attach to the targeting component without affecting its interaction with the targeted biochemical process (5). In studies of atherosclerosis, unique molecular imaging agents have been developed for the assessment of a wide variety of processes that contribute to atherogenesis. These have included the targeting of vascular cell adhesion molecule-1, monocyte recruitment, macrophage phagocytic activity, apoptosis, oxidative stress, matrix metalloproteinases, intraplaque hemorrhage, and neoangiogenesis (26–28). Numerous imaging modalities are used for the identification of these imaging agents including PET, single-photon emission CT, MRI, and ultrasound, as well as optical imaging modalities including bioluminescence and fluorescence that can be used for *in vivo* animal imaging. Of these the most promising at a clinical level are MRI and PET.

## Non-Molecular Imaging of Thrombosis

### IVUS and Combined OCT-IVUS Imaging of Thrombosis

IVUS, as the first intravascular imaging device, was introduced by Yock et al. in the 1980s (29) wherease, the OCT was introduced a few years later in the 1990s (30, 31). Although the two devices have the same basic principles and visualize the intracoronary structures by reconstructing images from signal waves scattered back from the vessel wall to the catheter, the utilized signals are different: ultrasound (wavelength 40–50 μm) in IVUS and low-coherence light (wavelength 1.3 μm) in OCT (32, 33). IVUS has been widely utilized as an auxiliary imaging modality with coronary artery angiography (CAG) to uncover the underlying mechanisms driving atherosclerotic diseases in clinical and research settings (34). IVUS yields cross-sectional and vertical axis images of the vascular walls and lumen, enabling the accurate assessment of thrombosis and atherosclerosis based on the quantification of luminal area, vessel size, non-protruding plaques, thrombosis, calcification, and the degree of positive vascular remodeling and plaque burden. Traditional grayscale IVUS images, however, are of relatively low resolution (axial resolution of 150–200 μm), restricting their utility as a tool for the evaluation of thrombosis (35). These IVUS images are particularly limited in their ability to detect acute and non-obstructive thrombi with high erythrocyte concentrations and low fibrin deposition. Efforts to improve IVUS imaging resolution to date have included virtual tissue IVUS (VH-IVUS) and integrated backscatter IVUS (BS-IVUS), enabling more reliable analyses of the characteristics of plaque composition (36). IVUS alone, however, is not well-suited to the diagnosis of thrombosis or the prevention of major adverse cardiovascular events (MACEs) owing to its poor resolution. To achieve optimal imaging, IVUS requires about 30 frames per second (fps), whereas OCT can be conducted at over 100 fps. The increases in IVUS imaging speed result in poorer image quality while decreasing OCT imaging speed prolongs the procedure and can thus increase the risk of procedure-related complications or misdiagnosis as a consequence of catheter spasm (37). Li et al. (38) designed a miniature integrated OCT-IVUS probe wherein an OCT lens and an IVUS transducer were placed back-to-back within a catheter, thus enabling confocal OCT-IVUS imaging in concert with an enhanced ultrasound transducer and a powerful graphical processing unit. Their resultant OCT-IVUS imaging system was able to capture images at 72 fps, enabling the visualization of 7 cm of the artery in 4 s. Recently developed multimodal intravascular imaging approaches including OCT-IVUS, multi-frequency IVUS, IVUS-NIRF, IVUS-IVPA, IVUS-FLIM/TRFS, and OCT-IVUS Fluorescence thus represent powerful approaches to improving image resolution and penetration depth, thereby aiding in the diagnosis of thrombosis in atherosclerosis patients (39). Future research has the potential to further improve IVUS imaging speeds and to unify OCT and IVUS technologies. IVUS and OCT are not well-suited to the assessment of plaque characteristics when used individually owing to limitations of resolution and penetration depth, but when used in combination with one another they effectively facilitate complementary intravascular imaging.

### OCT Imaging of Thrombosis

**Figure 2:**
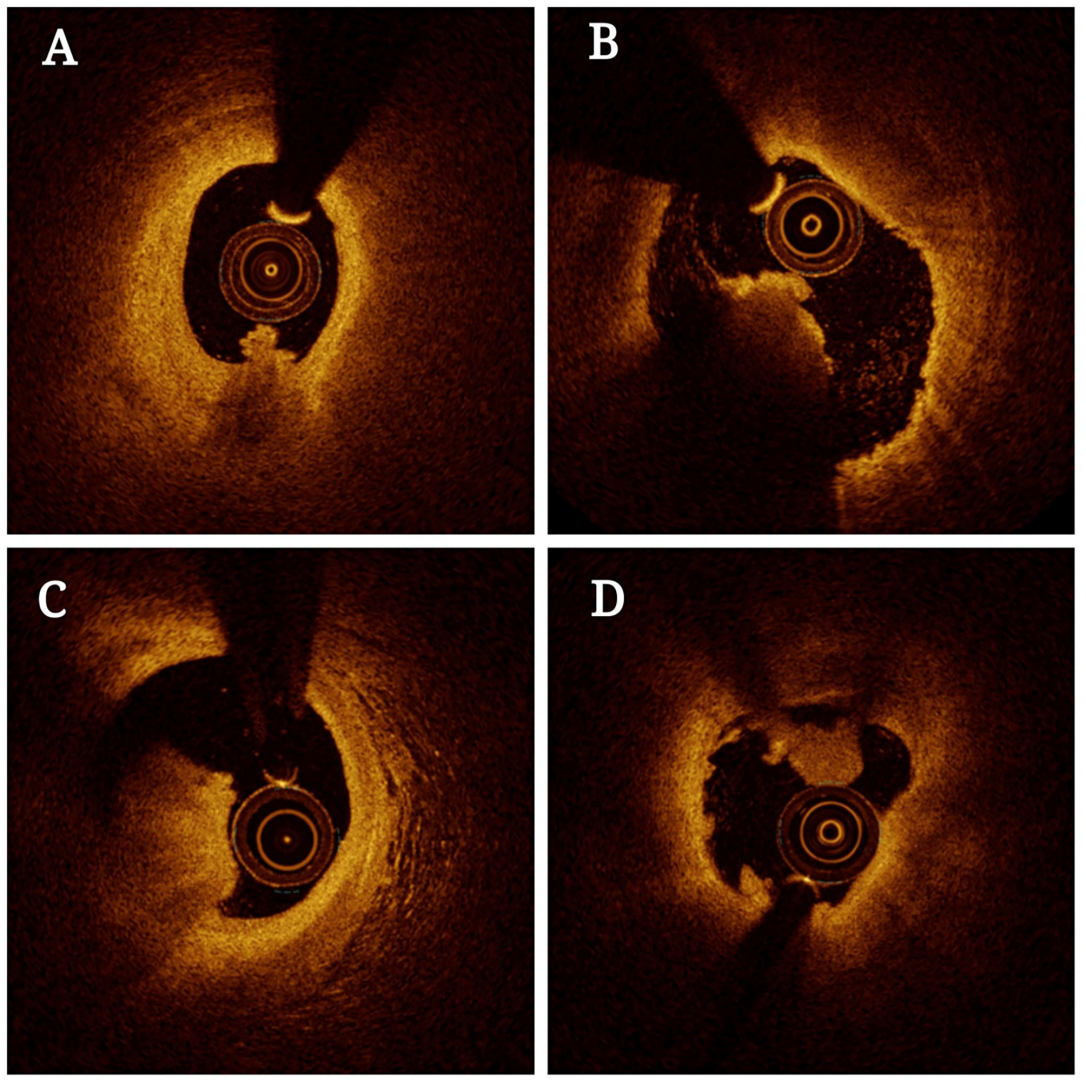
Optical coherence tomography (OCT) of thrombosis in atherosclerosis patients. The presence of residual red thrombus limits the visualization of plaque morphology **(A–C)**. OCT images in the distal and proximal segments of the thrombotic lesions exhibit an absence of superficial lipids or calcification **(B,D)**.

OCT is a powerful approach to the evaluation of coronary thrombosis and is currently an effective means of assessing vulnerable plaques (40). Coronary thrombi are characterized by OCT as irregular masses protruding into the vessel lumen discontinuous with the surface of the vessel wall and associated with ruptured or eroded plaques. White thrombi (platelet-rich) are identified as a homogeneous signal-rich mass with low-backscattering attenuation, while red thrombi (red blood cell-rich) are described as irregularly-shaped signal-free protrusions with high-backscattering attenuation (41). In this context, OCT is capable of evaluating plaque components and accurately detecting the presence of thrombi. Based on OCT features, coronary thrombosis is classified as either exhibiting extensive red thrombi with ruptured plaques that do not permit the assessment of vessel and plaque morphology or exhibiting a small number of white thrombi with eroded plaques underneath. Thrombosis with a normal endothelial lining underneath may be indicative of erosion (42). The prospective multicenter OCT in the assessment of coronary artery disease comparison with IVUS (OPUS-CLASS) study explored the reliability of frequency-domain optical coherence tomography (FD-OCT) with quantitative coronary angiography (QCA) and IVUS, and both that both FD-OCT and IVUS yielded more reproducible findings in a 100-patient cohort. IVUS, however, was associated with measured value variability twice that observed for FD-OCT (43). OCT also has an axial resolution of 10–20 μm and a lateral resolution of 30 μm, which is significantly higher than that of IVUS when generating high-resolution cross-sectional images (44). Thrombi in OCT images can be categorized as high-backscattering thrombi with a signal-free shadowing (red thrombi; erythrocyte-rich) or low-backscattering thrombi (white thrombi; thrombocyte-rich) (45). As OCT exhibits limited tissue penetration and is constrained by attenuation effects, however, thrombus images can become unclear, particularly for red thrombi, thus preventing thorough quantitative analyses of thrombi or underlying plaques, especially in the presence of lipid plaques or thrombus. Besides, OCT is not suited to visualization as far as the outer membrane (Figure 2) (46). Kaivosoja et al. (47) thus designed a novel computer image analysis-based approach to assessing thrombus morphology in OCT images. The backscatter, attenuation, and intensity values of OCT images generated using this algorithm were compared to standard consensus thrombotic type classifications generated by two independent analysts, demonstrating that this algorithm was a feasible approach to objectively overcoming the variability associated with subjective interpretations of OCT findings. In the TOTAL-OCT sub-study of the TOTAL trial (48), OCT was employed to quantitatively assess thrombi prior to stent implantation following the restoration of TIMI 2–3 flow to affected vessels. This analysis revealed that OCT-mediated measurements of the pre-stent thrombus burden were feasible and reproducible for patients suffering from ST-segment elevation myocardial infarction (STEMI). Intravascular imaging enables clinicians to provide insight that can help prevent coronary thrombosis by offering insights regarding plaque vulnerability. Nevertheless, due to existing imaging technical limitations and limited prospective data, more accurate approaches to predicting coronary thrombosis in atherosclerosis remain to be developed.

### CT Angiography Imaging of Thrombosis

CTA has been utilized as a means of detecting thrombosis in the left atrium/left atrial appendage (LA/LAA). However, it yields variable sensitivity and predictive value (NPV) with substantial variability among studies (49). Different cardiac CTA approaches such as two-phase scan cardiac CTA and dual-enhanced cardiac CTA yielded superior performance in the context of diagnosing thrombosis. These approaches, however, necessitate exposure to higher doses of contrast agents and radiation, limiting them from a technical perspective (50). Two-phase scan cardiac CTA protocols utilizing early-phase scans aimed at assessing intracardiac thrombi and late-phase scans capable of distinguishing thrombi from circulatory stasis have been developed, achieving markedly improved diagnostic specificity (98%) while retaining excellent NPV and sensitivity values. Dual-enhanced cardiac CTA protocols involving a single scan and two separate boluses of contrast agent injections have also been tested, with scans being performed a single time on a delayed phase 180 s following the administration of the first contrast bolus (51). CT Calcification Score (CAC) and Coronary Artery CTA (CCTA) can be used to directly image atherosclerotic plaques to more reliably determine the plaque burden. CCTA is associated with several advantages, including its ability to directly evaluate plaque morphology and to detect obstructive stenosis (52). Unlike CAC, CCTA can facilitate the recognition of calcified plaques, non-calcified plaques, thrombosis, and plaque characteristics. These prior data strongly suggest that CCTA is a powerful tool for the detection of atherosclerotic thrombosis. While CTA image quality has improved significantly in recent years, the spatial resolution of this approach remains limited. Future research is thus necessary to improve CTA imaging to reduce artifacts and decrease radiation doses. Further, coupling CTA with other imaging modalities will continue to improve image quality and guide the analysis of thrombosis in atherosclerosis patients.

### Cardiovascular Magnetic Resonance Imaging of Thrombosis

CMR is an imaging modality that can be used for the early detection of thrombosis in atherosclerosis patients, enabling risk stratification, and long-term monitoring (53). MRI can be used in the context of coronary and non-coronary angiography with or without contrast agents to facilitate flexible analyses of the features of endovascular stenosis and vulnerable plaques including intraplaque hemorrhage, thrombosis, lipid pools, macrophages, and thin-fiber-cap atherosclerotic plaques (54). MR angiography can facilitate the detection of coronary stenosis and thrombosis by using different T_1_ and T_2_ weights. Magnetic resonance sequences are a series of different radio-frequency (RF) pulses, applied at particular times in a specified way to obtain an image (55). Two key parameters that influence the image contrast are TR (repetition time) and TE (echo time). TR is the time interval between the application of an RF excitation pulse and the start of the next RF pulse, and TE is the time elapsed between the RF pulse and the peak echo which is detected (56). T1-weighted (T1W) sequences have both a short TR and TE. On T1W images fat, subacute hematomas, slow-moving blood, and gadolinium-based MR contrast appear bright, and tissues with fluid [water, cerebrospinal fluid (CSF), etc.] appear dark. T2W sequences have both a long TR and long TE. Free fluid and tissues with high free water appear bright (57). Using a variety of pulse sequences can not only allow for the detection of coronary artery stenosis but also provide insights pertaining to thrombus and plaque composition (58). Coronary arteries can be displayed by using a T_1_-weighted 3D gradient echo, and MR angiography can be used to distinguish thrombotic components in standard clinical field strength for 1.5–3T of T_1_-weighted images (59–61). This approach utilizes the correlation with high T_1_ signals associated with hemoglobin, which is a key component of fresh thrombi, allowing for the detection of thrombi as a bright high-signal region in T_1_-weighted 3D gradient-echo images. T_1_-weighted imaging can thus identify intraluminal plaque hemorrhage and thrombosis. T_2_-weighted imaging is sensitive to deoxygenated hemoglobin and ferrihemoglobin, and can thus also be used for thrombosis detection and evaluation (62). Currently, there are several limitations associated with CMR including a need to overcome artifacts associated with cardiac and respiratory movements, irregular movements and pulsation of the vascular vessels, and the difficulty in suppressing blood (63). Despite concerns pertaining to its validity as a tool for the characterization of thrombosis and vulnerable atherosclerotic plaques, CMR still offers promise and is widely studied in this context. There is thus a need to develop special sequences for the imaging of thrombi in atherosclerosis patients, and future work is required to understand the unique contrast, magnetic field strength, imaging feasibility, and other characteristics of these sequences. With future development, however, CMR has clear value as a non-invasive means of enabling the identification of endovascular stenosis, plaque burden, high-risk plaque features, and thrombosis.

### Angioscopy and Angiography of Thrombosis

Angioscopy is an intravascular visual imaging modality that can be used to directly visualize the vascular surface and to thereby study atherosclerotic structures. Angioscopy has also been used for the prediction of ACS events, thrombus formation, and analyses of plaques following the deployment of drug-eluting stents (64). Savastano et al. (65) developed a high-resolution multimodal scanning fiber angioscopy (SFA) approach enabling the direct visualization of the intraluminal vascular surface to detect subtle thrombotic ulcers or other lesions in non-stenotic atherosclerotic plaques or the context of low-grade arterial stenosis. Coronary angiography is also a highly specific approach to intravascular thrombus detection. Amraotkar et al. (66) explored the use of coronary angiographic characteristics (spherical, ovoid, or irregular filling defects, abrupt vessel cutoff, intraluminal staining, and any coronary filling defects) for the identification of acute coronary thrombi in 80 patients suffering from acute myocardial infarction or stable coronary artery disease. They ultimately found that spherical, ovoid, or irregular filling defects, as well as intraluminal staining, were predictive of the presence of a coronary thrombus. If these findings can be validated in a separate patient dataset, they will offer key value in the diagnosis of acute coronary thrombosis. However, it is important to note that other conditions such as pseduothrombi associated with aneurysm, embolism, coronary dissection, or mural calcification can also present with similar angiographic findings (67). As such, additional multimodal approaches to thrombus imaging in patients with atherosclerosis can provide complementary insights with key therapeutic implications. The studies discussed above provide evidence that angioscopic and angiographic approaches are a valuable means of diagnosing thrombosis in those suffering from atherosclerosis.

## Molecular Imaging of Thrombosis

### Ultrasound Molecular Imaging of Thrombosis

Ultrasound molecular imaging relies on the delivery of specific contrast agents to particular vascular regions as a means of assessing the expression of biomarkers of thrombosis, and the prerequisite for ultrasound molecular imaging is the availability of contrast agents (68). Multiple ultrasound contrast agents (UCAs) have been developed, to date, including micro-bubbles (MBs), nanobubbles (NBs), phase change contrast agents (PCCAs), and echogenic liposomes (ELIPs) (69). These UCAs are effective tools for the characterization of thrombosis in atherosclerosis by analyzing biomarkers of thrombosis. Microbubble-based UCA has been approved by the federal Food and Drug Administration (FDA) for various clinical applications. UCA can substantially improve the diagnostic abilities of not only echocardiography but also of ultrasound of the liver, kidney, and other organs (70–72). On the other hand, of all the nanoparticles, liposomes are a prime candidate for drug delivery because of their structural similarities with biological cells, long circulation times, and ability to carry both hydrophobic (73) and hydrophilic (74, 75) drugs. Echogenic liposomes combine these advantages of liposomes with the echogenicity or ultrasound responsiveness of microbubbles, making them an excellent candidate for concurrent ultrasound imaging and drug delivery (76). Several groups have demonstrated that micro-bubbles (MBs) exhibit excellent affinity for GPIIb/IIIa receptors, and relative to low-boiling micro-bubbles (MBs) and nanobubbles (NBs), MB-specific imaging is more sensitive, and contrast provided by MBs is greater than that provided by NBs, thus providing a wealth of insight pertaining to the molecular and cellular basis of thrombosis including deposition of activated platelets, fibrin, and tissue factors (77). Notably, investigated recently, targeted therapeutic micro-bubbles (TT-MBs) were shown to be effective for ultrasound-based diagnostic molecular imaging of thrombosis and thrombolytic therapy (78). Wu et al. (79) applied arachidonic acid (AA) to the bilateral carotid arteries of mice to induce thrombosis, and then conducted micro-bubble (MB)-Glutamic acid cyclic peptide (cRGD) ultrasound imaging of the bilateral carotid arteries in these mice via targeting activated GPIIb/IIIa receptors. Mounting evidence has shown that MB-cRGD has an excellent affinity for activated GPIIb/IIIa receptors under conditions of variable shear stress *in vivo* (80). Novel dual-ligand MBs have also improved MB contrast agent target binding activity, using anti-LIBS and the selectin ligand siayl lewis^a^ to enhance MB capture ultimately leading to effective MB adherence to activated platelets even under shear stress conditions *in vitro* (81). Wang et.al have demonstrated that glycoprotein IIb/IIIa–targeted MBs specifically bind to activated platelets *in vitro* and allow real-time molecular imaging of acute arterial thrombosis and monitoring of the success or failure of pharmacological thrombolysis *in vivo* (82). In one recent study, researchers conjugated antibody scFv fragments with anti-LIBS and scuPA to generate activated-platelet-specific targeted ultrasound diagnostic MBs (TT-MBs) that could be used to diagnose thrombosis and to monitor outcomes associated with fibrinolysis and thrombolytic therapy (83). In this way, these targeted TT-MBs are specifically bound to the GPIIb/IIIa receptors on activated platelets, allowing researchers to conduct molecular imaging of thrombi. Thrombus-targeted ultrasound contrast agents are key determinants in the context of molecular imaging via ultrasonography enabling the detection of MBs or certain other targeted nanoparticles including liposomes, polymer nanoparticles, and metal nanoparticles, micelle, perfluorocarbon nanoparticle, synthetic lipoprotein particle, polymer-derived microparticle, and carbon nanotube (84). As an alternative approach, Yan et al. (83) developed vascular cell adhesion molecule-1(VCAM-1)/Intercellular Adhesion Molecule 1(ICAM-1)/P-selectin-specific MB_VIS_ via preparing MBs with surfaces that had been coated with synthetic polymeric sialyl Lewis X (sLe^x^) and with antibodies specific for VCAM-1 and ICAM-1. Relative to single-or dual-targeted MBs their MB_VIS_ preparations were far more sensitive for the ultrasonographic imaging of atherosclerotic progression because they employed the strategy of mimicking leucocytes that are recruited to the vessel wall during the initiation of atherosclerosis through selectin-dependent arrest and cell adhesion molecule-mediated firm cell adhesion. As discussed in the above studies, these contrast agents can be effectively used to facilitate ultrasound molecular imaging of thrombosis in atherosclerosis patients, offering insight into thrombus composition (fibrin, platelets, erythrocytes, cholesterol crystals, and leukocytes contents). There remains great potential for the further development of this imaging modality and associated contrast agents to further improve thrombosis diagnosis and treatment.

### Magnetic Resonance Imaging of Thrombosis

The use of contrast agents has increased the sensitivity and specificity of MRI. The contrast in MRI is multifactorial, depending not only on T1 and T2 relaxation rates, but also on flow, proton density, and, in gradient-echo sequences, on the angle of the induced field. The use of contrast agents in MRI changes the T1 and T2 relaxation rates, producing increased signal intensity on T1-weighted images or decreased signal intensity on T2-weighted images, or both. All contrast agents produce changes in magnetic susceptibility by enhancing local magnetic fields. These effects are caused by interactions between nuclear and paramagnetic substance magnet moments, which produce accentuated transitions between spin states and cause shortening of T1; the paramagnetic substance causes accentuated local fields, which lead to increased dephasing and thus shortening of T2 or T2^*^ relaxation time. The efficacy of shortening of T1, T2, or T2^*^ relaxation time depends on the distance between the proton-nucleus and the electronic field of the paramagnetic compound, the time of their interaction (correlation time), and the paramagnetic concentration. The MRI contrast agents currently in use cause shortening of T1, T2, or T2^*^ relaxation time. Metal chelates [e.g., gadolinium-diethylene triamine penta-acetic acid (Gd-DTPA)] in low concentration cause shortening of T1 relaxation times, and the superparamagnetics (e.g., ferrite) cause shortening of T2 relaxation times (85). Thrombus-targeted MRI can facilitate the identification of thrombi in the context of atherosclerotic plaques, with particular imaging targets enabling radiographic differentiation between newer active thrombi and older thrombi (86). Several MR contrast agents with shortening ability of T1, T2, or T2^*^ relaxation times, a long half-life, and satisfactory safety profiles have been developed including Gd-DTPA, superparamagnetic iron oxide (SPIO), and ultra-small superparamagnetic iron oxide particles (USPIOs) (87). In one recent report, researchers designed ultra-small magnetic dual contrast iron oxide nanoparticles (DCIONs) that had been functionalized via conjugation to activated platelet-specific scFv antibody fragments. The binding activity of the scFv antibody fragments was retained and resulted in the successful targeting of contrast agents to thrombosis as demonstrated *in vitro* and *in vivo* experiments (88). In another study (89), Gd-DTPA polylactic acid copolymeric glycolic acid (PLGA) nanobubbles were designed that exhibited superior thrombus targeting, longitudinal relaxation time (T_1_), and imaging utility. Research has also shown the development of novel Fe_3_O_4_-based PLGA nanoparticles (Fe_3_O_4_-PLGA-cRGD) to be a feasible strategy for MRI-based molecular thrombus imaging (90). Perfluorocarbon (PFC) nanoparticles have also been used by several groups to image thrombi and to treat early thrombosis via the delivery of perfluorocarbon nanoparticles combined with the thrombin inhibitor D-phenylalanyl-L-prolyl-L-arginyl-chloromethyl ketone (PPACK) into the carotid artery in a murine model of acute thrombosis (90). When coupled with PPACK, these perfluorocarbon nanoparticles offer value as tools for both molecular imaging of active thrombosis and localized antithrombotic/thrombolytic treatment at sites of active clotting without causing significant side effects (91). By loading thrombus-targeted nanoparticles with high concentrations of both imaging agents and thrombolytic drugs, researchers can utilize this targeted delivery platform to minimize any potential systemic side effects while enabling the real-time imaging of thrombi and the monitoring of thrombolytic therapeutic efficacy. Photoacoustic (PA)/Magnetic resonance (MR) dual-modality nanoparticles (PA/MR-NPs) with dual ligands were successfully constructed via the double-emulsion method and the pDA method. PA/MR bimodal nanoparticles have been used to target mixed thrombi under high shear stress *in vivo* for both imaging and thrombolytic treatment in one example of such a dual-modal platform (92). These targeted nanoparticles exhibit good thrombus affinity, and passive targeting or surface modifications can be used to achieve appropriate contrast for thrombus imaging or drug delivery in a ligand-specific manner (93). Thrombus-targeting nanoparticles combined with high-concentration of thrombolytic drugs and imaging agents can be delivered to the targeted site, thereby reducing systemic side effects, providing real-time imaging of thrombus and information regarding the efficacy of thrombolytic therapy (94).

^19^F MRI has emerged as a promising novel technique for molecular imaging. For this, emulsified, biologically inert PFCs are used as a contrast agent to follow the fate of *ex vivo* or *in vivo* PFC-labeled cells (95–97). Because ^19^F is physiologically found in biological tissue in only trace amounts, the resulting fluorine signal displays an excellent degree of specificity. The merging of ^19^F images with corresponding ^1^H data sets enables the exact anatomic localization of the ^19^F signal. Temme et al. reported a novel technique for the sensitive and specific identification of developing thrombi using background-free ^19^F magnetic resonance imaging, together with α2-antiplasmin peptide (α2^AP^)–targeted perfluorocarbon nanoemulsions (PFCs) as contrast agent, which is cross-linked to fibrin by active factor XIII (98). Their results demonstrated that ^1^H/^19^F magnetic resonance imaging, together with α2^AP^-PFCs, is a sensitive, non-invasive technique for the diagnosis of acute deep venous thrombi and pulmonary thromboemboli. Furthermore, ligand coupling by the sterol-based postinsertion technique represented a unique platform for the specific targeting of PFCs for *in vivo* ^19^F magnetic resonance imaging. Wang et al. recently established an antibody-targeted, ^19^F-based MRI approach that enables selective imaging of activated platelets (86). They used the human single-chain antibody (scFv) conjugated biotin-lipid scFv-LIBS to visualize thrombi within the inferior vein by 1H/19F MRI. These prior studies have shown that by developing novel MR contrast agents with a long half-life, a high relaxation rate, specific targeting capabilities, and minimal side effects, it is possible to enhance the specificity and resolution of molecular MRI-based thrombus imaging. We anticipate that MRI approaches to the detection of thrombosis in atherosclerosis patients will continue to develop, providing a simple, effective, and reliable molecular imaging modality.

### Radionuclide Imaging of Thrombosis

At a simplified level, radionuclide imaging techniques such as PET and SPECT rely on the injection of radioactive nuclide markers and the subsequent use of imaging equipment to visualize them *in vivo*, thus allowing for the non-invasive monitoring of cellular metabolism and function at the molecular level. SPECT and PET approaches have been employed in recent years to evaluate thrombosis and to assess the molecular processes active within vulnerable plaques (99, 100). Several studies have utilized radionuclide imaging approaches to analyze different thrombosis-related molecular markers. For example, Andrews et al. (15) developed ENC2015, which is a novel factor XIIIa-directed optical and PET radiotracer to image thrombosis *in vivo* in a non-invasive fashion. This radiotracer was designed using the small SPECT factor XIIIa-targeted ^99M^TC-NC100668 radiotracer. It enables thrombus detection before any clinical manifestations thereof in addition to facilitating longitudinal monitoring of thrombus activity and the efficacy of thrombolytic therapeutic efficacy. Senders et al. (101) explored the utility of the novel ^89^Zr-LA25 PET probe capable of targeting malondialdehyde-acetaldehyde epitopes, and found that it was able to specifically localize to ruptured atherosclerotic plaques exhibiting accompanying thrombosis. Clinical work has highlighted the development of the PET tracer ^18^F-GP1, which is a novel tracer with a high affinity for GPIIb/IIIa on platelet surfaces (102). Following ^18^F-GP1 injection, PET/CT researchers were able to monitor this tracer in a dynamic and system-wide manner, while analyzing plasma samples to assess ^18^F-GP1 clearance and metabolism (103). They found that ^18^F-GP1 exhibited satisfactory biodistribution and pharmacokinetic properties that made it ideal for the detection of atherosclerotic thrombosis. Oliveira et al. (104) sought to enhance the radionuclide-mediated imaging of thrombosis by developing fibrin-binding peptides coupled to different radioactive tracers (^68^Ga, ^111^In, or ^99^mTc) which were able to specifically interact with arterial thrombi in a rat model of thrombosis when assessed via multimodal SPECT/PET imaging. Blasi et al. (105) showed that the fibrin-binding peptides labeled with ^64^Cu could detect multiple thrombi following a single systemic PET scan. Uppal et al. (106) applied EP-2104R and ^64^Cu to conduct dual PET/MR imaging as an approach to detecting thrombi within the carotid arteries of experimental rats, while Heidt et al. (107) utilized ^111^In-labeled LIBS capable of specifically binding to activated platelet for use in carotid artery thrombosis SPECT/CT imaging. Ardipradja et al. (108) similarly employed an ^18^F-labeled LIBS probe to conduct PET/CT imaging of the carotid artery in a murine thrombosis model. Both PET and SPECT imaging target molecules exhibit excellent sensitivity from a clinical perspective, but these traditional radionuclide-based imaging modalities exhibit relatively poor spatial resolution (SPECT 5–8 mm^3^; PET 3–5 mm^3^). Modern SPECT or PET scanning instruments are therefore combined with high-resolution MRI or CT instruments to facilitate hybrid imaging (SPECT/CT, PET/CT, PET/MR), thereby achieving highly sensitive high-resolution functional spatial imaging capable of localizing thrombi in the context of atherosclerosis (109). As these prior results emphasize, radionuclide imaging can enhance the detection of gamma rays following the injection of radiopharmaceuticals to generate 3D physiological images that can be employed for thrombus molecular imaging through a range of different strategies.

### Optical Imaging of Thrombosis

Optical imaging is a form of molecular imaging that utilizes powerful optical microscopy equipment in combination with different optical contrast mechanisms (such as optical absorption, backscatter, scattering, and endogenous or exogenous fluorescence). Optical fluorescence imaging using a range of reagents such as fluorescently labeled antibodies, fluorescently labeled ligands specific for thrombosis-related targets, fluorescently labeled platelets, and reagents that fluoresce in response to thrombin activation have been used to optically assess thrombosis (110). Kwon et al. (111) developed protease-sensing near-infrared fluorophore molecular imaging agents to detect VCAM-1 expression by cells in atherosclerotic lesions, and utilized an intravital fluorescence microscope to capture FXIIIa-related near-infrared fluorescence (NIRF) signal due to the reduced expression of VCAM-1 *in vivo*. Results of their study demonstrated that these reagents were suitable for the visualization of FXIII coagulation enzyme activity as a means of monitoring thrombus age, which was proportional to FXIIIa-related NIRF signal intensity. Similarly, Lim et al. (112) utilized a novel 3D fluorescence emission tomography (FLECT) approach to the detection of arterial thrombosis in mice, while using a novel activated platelet-specific fluorine probe (Targ-Cy7) containing a single-chain antibody-fragment (scFv_Targ_) and coupled to the near-infrared (NIR) Cy7-dye as a means of detecting atherosclerosis. This FLECT strategy was successfully utilized to detect sites of thrombosis *in vivo* due to the specific detection of the fluorescent signal in the murine artery. Stein-Merlob et al. (113) designed novel NIRF ultra-small superparamagnetic iron oxide (USPIO) nanoparticles (CLIO-CyAm7) that specifically accumulated in areas of atheroma in a rabbit model of atherosclerosis, thereby facilitating combination imaging. Moreover, Bonnard et al. (114) created PASKE particles composed of proline, alanine, and serine (PAS) cross-linked with lysine (K) and polyglutamic acid (E) using mesoporous silica templates, and labeled these particles with NIRF molecules. In a rat thrombosis model system, these PASKE particles are specifically bound to GPIIb/IIIa on activated platelets, thus facilitating arterial thrombosis detection. Together, these prior findings highlight the value of optical imaging modalities to gain molecular and cellular insights into the mechanistic basis for atherosclerotic thrombosis.

### Multimodal Imaging of Thrombosis

It is essential to select appropriate application-specific molecular imaging modalities in light of the relative advantages and disadvantages of each approach. A given modality may be insufficient to obtain all of the required information on a given target or process of interest, and each is associated with a range of limitations associated with the spatiotemporal resolution, depth of subject penetration, and detection threshold (88) (Table 2). Optimal fluorescence approaches, for example, are high-resolution strategies that are very sensitive and specific, but they are constrained by limited tissue penetration, precluding their use for the imaging of deep tissue targets (115). SPECT, PET, and related modalities, in contrast, allow for the highly sensitive visualization of metabolic and physiological processes but offer relatively poor spatial resolution (116). Combination imaging strategies such as PET-CT or SPECT-CT scanners can provide complementary and supplemental information pertaining to morphology and function that is relevant in a clinical context, making them superior to unimodal imaging in most contexts (117). In recent years, researchers have explored the utility of SPECT-CT, IVUS-intravascular photoacoustic (IVPA), IVUS-fluorescence lifetime imaging (FLIM), IVUS-intravascular (IVOCT), and IVOCT-NIRF combination approaches to the high-specificity detection of plaque structure and composition (118). Moreover, the development of thrombosis-specific molecular imaging agents to detect and monitor thrombogenesis and fibrinolysis *in vivo* have positive outcomes to improve the diagnosis, risk stratification, and treatment of thrombosis syndromes. In an example, McCarthy et al., synthesized efficient multimodal nanoagents (CLIO-GPRPP, CLIO-FXIII) targeted to two different constituents of thrombi, namely, fibrin and activated factor XIII. These agents are targeted via the conjugation of peptide-targeting ligands to the surface of fluorescently labeled magnetic nanoparticles. As demonstrated by *in vitro* and *in vivo* studies, both nanoagents possess high affinities for thrombi, and enable multimodal fluorescence and magnetic resonance imaging (119). The coupling of PET and MR technologies can overcome many of the resolution-based limitations of PET and SPECT, making these combined PET-MR and SPECT-MR imaging modalities ideal for the identification of thrombi in the context of atherosclerosis *in vivo* by enhancing the signal, decreasing contrast agent dose, and reducing scanning time when using an injected multimodal probe. Supplementary morphological and functional data derived from these PET/SPECT-MR scans can thus aid in surface marker-based detection of atherosclerotic thrombi (116). Future work has the potential to better integrate these different imaging modalities in a manner that overcomes their limitations, further aiding efforts to characterize and detect thrombosis *in vivo*. Owing to their convenience and comprehensive nature, multimodal molecular imaging strategies represent an important direction for research regarding the accurate diagnosis of early thrombosis in atherosclerosis patients.

**Table 2:** Comparative overview for different imaging modalities.

**Imaging modality**	**Ultrasound**	**MRI**	**PET SPECT**	**Optical imaging (fluorescence)**	**IVUS**	**OCT**	**Angiography**
Emission	Sound waves	Radio waves	Gamma rays	Near-infrared light	Sound waves (Intravascular)	infrared light	X-rays
Wavelength (μm)	35–80	NA	<0.01	0.8–2.5	35–80	1.3	<0.01
Penetration (mm)	10.0	0.25	>10.0	1.0–2.0	10.0	1.0–2.5	0.0
Resolution (μm)	NA	100	400–600	NA	100–200	Axial: 10–20lateral: 30	>500
Thrombus imaging	+	++	++	+	+	+++	++
Advantages	Low cost, high sensitivity, early quantitative evaluation, no trauma, no radioactivity, and high safety, real-time imaging	Non-invasive, non-ionizing radiation, high spatial resolution, deep tissue penetration and excellent soft-tissue contrast, functional-anatomical imaging	High sensitivity, large number of molecular probes, quantitative evaluation, multiple isotopes, physiological imaging	Low cost, high sensitivity, no radioactivity, multiple molecular probes, portable and real-time imaging	The real-time assessment of vessel wall and plaque components (lipid core, thrombus), cross-sectional and vertical axis imaging	The evidence of OCT used for evaluation of coronary artery thrombosis is considered very high, high-resolution cross-sectional imaging	high specificity, real-time assessment of lesion, vascular stenosis, widely used in clinical and research applications
Disadvantages	Limited field of view	Limited contrast agents	Low spatial resolution, radiation, expensive	Low tissue penetration	Limited imaging resolution	Low tissue penetration, the attenuation effect	Radiation, invasive, lack sensitivity

*Imaging modalities for assessing thrombosis in atherosclerosis have their strengths and drawbacks with respect to spatial and temporal resolution, depth of subject penetration, and detection threshold. Therefore, appropriate imaging technology selection requires sufficient consideration of each method's advantages and disadvantages. MRI, magnetic resonance imaging; PET, positron emission tomography; SPECT, Single-photon emission computed tomography; IVUS, intravascular ultrasound; OCT, optical coherence tomography*.

## Conclusion and Future Perspectives

**Figure 3:**
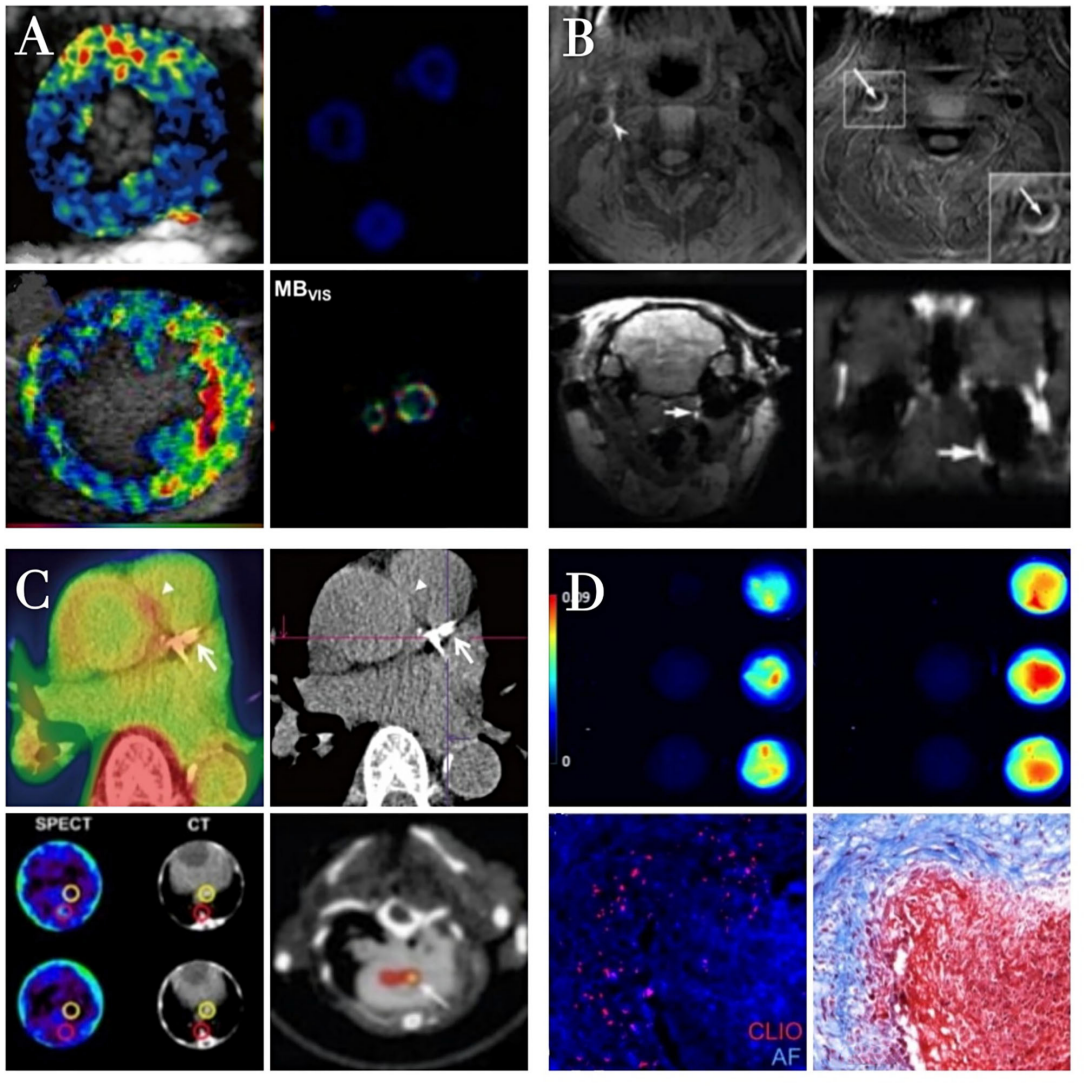
The characteristics of different molecular imaging approachs. Ultrasound molecular imaging of thrombosis **(A)**. Color-coded ultrasound molecular signal overlays indicate that targeted MBs yield a specific signal only in the presence of thrombin. Reproduced with permission from Yan et al. (83). Copyright Theranostics. 2018. Magnetic resonance imaging of thrombosis **(B)**. The high MR signal area of thrombi is significantly increased after contrast agent injection in the presence of atherosclerotic plaques Reproduced with permission from Zhang et al. (92). Copyright Int J Nanomedicine (2019). Radionuclide imaging of thrombosis **(C)**. Fibrin-binding peptides coupled to radioactive tracers specifically interacting with arterial thrombi as detected in PET/SPECT images. Reproduced with permission from Lee et al. (120). Copyright Korean J Radiol (2015). Optical imaging of thrombosis **(D)**. Fluorescence imaging of nanoparticles binding to fibrin clots formed from fibrinogen and thrombin. Reproduced with permission from Wen et al. (121). Copyright J Mater Chem B (2015).

As non-molecular and molecular imaging strategies for the imaging of atherosclerosis have evolved, analyses have transitioned from direct irradiation to more comprehensive multidirectional assessments of the lumen, vessel wall, thrombus, plaque burden, and vulnerability (Figure 3). Multimodal imaging can facilitate detailed three-dimensional structural and functional analyses of the coronary artery, allowing clinicians to diagnose atherosclerosis, monitor disease progression, and predict patient prognosis. Thrombosis in atherosclerosis patients typically occurs as a consequence of atherosclerotic plaque rupture. The major detection methods for thrombi include US, CTA, MRI, and digital subtraction angiography (DSA), which are effective for detecting clots in large vessels, but not useful for detecting microthrombi in coronary microvessels. Novel imaging modalities can facilitate the detection of plaque hemorrhage and rupture associated with poor patient prognosis. In the future, thrombosis imaging may become a standard approach to cardiovascular disease management when employed in concert with specific disease-related biomarkers that can aid in identifying high-risk plaques and diagnosing thrombotic diseases. Such imaging approaches will provide dynamic information pertaining to the progression of thrombosis in atherosclerosis patients, guiding their personalized treatment. Already, there has been much progress in the area of molecular imaging for vascular diseases. Yet the hurdles to translation into the clinical realm are considerable. Although ultrasound and MRI molecular contrast agents have been used in humans, of the modalities discussed above, only PET and SPECT have molecular contrast agents in routine clinical use. A challenge to scientists and their commercial partners is how to pick the right agent at the right time in its development and to define an application to move forward to the clinic.

The contrast clearance from the body is also worth mentioning and is an important consideration. In the first place, rapid blood phase clearance may be required to remove background signals to permit the identification of retained contrast at the site of interest. Afterward, clearance from the target site will permit repeated measures, for example, to monitor response to treatment. In the final phase, the agent should undergo disposal or excretion, with or without prior dismantling to component elements. Moreover, a favorable safety profile is a prerequisite for clinical use, and the regulatory requirements for demonstration of safety are no less stringent for diagnostic contrast agents than for therapeutic agents. Areas requiring evaluation include immunogenicity, radioactivity, chemical toxicity, the potential for pharmacological action, physical toxicity (e.g., vessel plugging), the potential for accumulation on repeated dosing. In general, little is known of these, and studies tend to be small scale, organ- or system-specific with a lack of dose-ranging evaluation, the effect of repeat administrations, time course, or anything other than immediate toxicology. As candidates for translation to human emerge, evaluation of these parameters will be vital.

Moreover, recent efforts in establishing and optimizing molecular imaging probes that target specific ingredients in the blood clots opened new horizons for the detection of thrombus and overcoming shortcomings of the existing techniques. Examples of these targets are GP IIb/IIIa cyclic RGD peptides and cyclic fibrin binding peptides. RGD also binds to other receptors, such as αvβ3, α5β1, and have been used for other diseases, including cancer. While RGD has a slightly higher affinity to activated platelet, it binds to both activated and inactivated platelets thus novel targets identification is also vital for successful translation of these approaches to clinic. Apcitide is the only FDA-approved agent among these tracers, which is in phase III multicenter study for DVT. Further studies using other modalities such as PET are suggested to evaluate the role of Apcitide in the detection of DVT. Another recently introduced agent investigated in multimodality MRI/PET/SPECT/NIRF studies is EP-2104R. However, only the MR modality has proceeded to human feasibility studies with positive results. Anti D-dimer DI-80B3 Fab' fragment (Thromboview) is another newly introduced agent which has shown favorable sensitivity and specificity for detection of suspected DVT and acute PE.

FDG-PET/CT has been able to detect thrombosis, mainly in the venous system anywhere in the body in patients with suspected DVT. Preliminary data suggest an acceptable sensitivity in the early, even in pre-symptomatic patients. It can also differentiate acute from chronic thrombi that are no longer active and eliminate the need for unnecessary treatment. Furthermore, accurate quantification with FDG-PET/CT allows monitoring response to treatment. As breakthroughs in the development of molecular and non-molecular imaging technologies are made, we predict that they will significantly enhance the prevention, diagnosis, and treatment of thrombosis in atherosclerosis patients.

## Author Contributions

BG designed and wrote the review, supervised the process, and critically reviewed the complete manuscript. ZL, PT, and HT performed the literature search and prepared the figures. YT revised the text and critically discussed the completed manuscript. All authors read and approved the final manuscript.

## Conflict of Interest

The authors declare that the research was conducted in the absence of any commercial or financial relationships that could be construed as a potential conflict of interest.

## Abbreviations

ACS: acute coronary syndrome
ADP: adenosine diphosphate
MRI: magnetic resonance imaging
PET: positron emission tomography
SPECT: single-photon emission computed tomography
CT: computed tomography
IVUS: intravascular ultrasound
OCT: optical coherence tomography
CTA: computed tomography angiography
CMR: cardiovascular magnetic resonance
UCAs: ultrasound contrast agents
MBs: micro-bubbles
NBs: nanobubbles
PCCAs: phase-change contrast agents
ELIPs: echogenic liposomes
TT-MBs: targeted therapeutic micro-bubbles
VCAM-1: vascular cell adhesion molecule-1
ICAM-1: intercellular Adhesion Molecule 1
AA: arachidonic acid
MB-cRGD: micro-bubble- Glutamic acid cyclic peptide
Gd-DTPA: gadolinium diethylenetriaminepentaacetic acid
SPIO: superparamagnetic iron oxide
USPIOs: ultra-small superparamagnetic iron oxide particles
DCIONs: ultra-small superparamagnetic iron oxide particles
PLGA: polylactic acid copolymeric glycolic acid
PPACK: D-phenylalanyl-L-prolyl-L-arginyl-chloromethyl ketone
PA/MR-NPs: photoacoustic/Magnetic resonance dual-modality nanoparticles
NIRF: near-infrared fluorescence
FLECT: fluorescence emission tomography
IVPA: intravascular photoacoustic
IVOCT: intravascular optical coherence tomography
FLIM: fluorescence lifetime imaging
CAG: coronary artery angiography
VH-IVUS: virtual tissue IVUS
BS-IVUS: integrated backscatter IVUS
MACEs: major adverse cardiovascular events
FD-OCT: frequency-domain optical coherence tomography
QCA: quantitative coronary angiography
STEMI: ST-segment elevation myocardial infarction
LA/LAA: left atrium/left atrial appendage
NPV: negative predictive value
PPV: positive predictive value
CAC: CT Calcification Score
CCTA: Coronary Artery computed tomography angiography
SFA: scanning fiber angioscopy.
